# Effect of the denture adhesive for dry mouth on the retentive force of the experimental palatal plates: a pilot controlled clinical trial

**DOI:** 10.1186/s12903-023-02983-3

**Published:** 2023-05-31

**Authors:** Kunihito Yamane, Yuji Sato, Junichi Furuya, Osamu Shimodaira

**Affiliations:** Division of Oral Function Management, Department of Oral Health Management, Showa University School of Dentistry, Ota-ku Tokyo, Japan

**Keywords:** Denture adhesive, Dry mouth, Palatal plate, Removable dentures, Retentive force

## Abstract

**Background:**

A denture adhesive for dry mouth with good cleaning properties has recently been developed. While previous studies on models have shown the effectiveness of denture adhesives in terms of retention and cleanability, no reports have evaluated their effectiveness in the oral cavity. The aim of this study was to compare and investigate the retention and usability of an experimental palatal plate in the dentulous jaw using a denture adhesive for dry mouth, a conventional cream-type denture adhesive, an oral moisturizer, and a denture moisturizer.

**Methods:**

Ten healthy dentulous participants (mean age 27.2 ± 1.6 years) were included in the study. Palatal plates were fabricated. Four test samples were used: denture adhesive for dry mouth, conventional denture adhesive (cream type), oral moisturizer, and denture moisturizer. The sample was applied to the inner surface of the palatal plates, and the retentive force of the palatal plate was measured every 10 min for 30 min. After the measurements, the study participants were asked to rinse the palatal plate with water and subjectively evaluate the samples used.

**Results:**

The conventional denture adhesive (cream type) showed increased retentive force over time, with the maximum retentive force obtained after 10 min of application. However, its washability was rated second lowest. The denture adhesive for dry mouth showed the highest retentive force immediately after application. Its washability was also good.

**Conclusions:**

The results suggest that the denture adhesive for dry mouth has reasonable retentive force in the oral cavity and cleaning properties compared to the conventional cream-type denture adhesive.

## Background

The proportion of older individuals in Japan has been increasing. According to the Statistics Bureau of the Ministry of Internal Affairs and Communications in 2022, the ageing rate was 29.3% and is expected to increase in the future [1]. Since tooth loss increases with age, the number of ageing patients with multiple missing teeth who require removable dentures are also more likely to increase in the future.

With the ageing of patients who require removable dentures, the oral cavity becomes more prone to oral motility disorders due to neurological diseases such as stroke and Parkinson’s disease [2, 3]. dry mouth due to drug side effects [4], and advanced jaw resorption [5]. In particular, dry mouth, which is common among older individuals, is often a clinical problem because it can lead to decreased retentive force of dentures and discomfort. These changes in the oral environment due to ageing and disease can make denture maintenance and stability difficult; thus, the use of denture adhesives may be beneficial. In fact, Nicolas et al. [6] reported that denture adhesives improved denture stability and oral health-related quality of life, especially in complete denture wearers with poor oral health-related quality of life.

However, denture adhesives tend to adhere and remain on the oral mucosa and denture base. The American College of Prosthodontists’ guidelines on denture adhesives have also mentioned its problems regarding cleanability [7]. Residual denture adhesives can promote the growth of oral bacteria and *C. albicans*, causing denture stomatitis [8], thereby increasing the risk of aspiration pneumonia in the older people [9, 10]. This risk is particularly high for older people requiring long-term care, as they often experience oral hygiene difficulties.

Furthermore, denture adhesives are water absorbent and may contribute to dry mouth. For this reason, oral moisturizers with good cleaning properties may be used as a substitute for denture adhesives in patients with dry mouth [11–13]. However, some problems were encountered with the use of oral moisturizers as denture adhesives. First, many oral moisturizers contain artificial sweeteners, which can spoil the flavour of food. Second, the low pH of some moisturizers may lead to the demineralisation of remaining teeth. Moreover, unlike denture adhesives whose safety as controlled medical devices has been confirmed by the Japanese Industrial Standards and International Organization for Standardization, oral moisturizers may not meet the required properties of denture adhesives.

A gel-type denture adhesive for dry mouth with good cleaning properties has been recently developed for denture wearers with dry mouth [14]. Ohno et al. [14] measured the retention of two resin plates by drying them and placing a sample between them. They found that denture adhesives for oral dryness showed significantly higher retention than cream-type denture adhesives or oral moisturizers. Furthermore, a study by Ikemura et al. [15], which measured the changes in retentive force over time on a model immersed in water, found that the denture adhesive for dry mouth showed higher retentive force than oral moisturizers during the first 30 min of measurement.

However, there have been no reports comparing the retentive force of dentures in the actual oral cavity with the use of denture adhesives, including those for dry mouth. This may be because denture wearers, especially edentulous patients, have various oral conditions in terms of the shape of the jaw crest, degree of oral dryness, etc. Moreover, measuring retentive force under the same conditions is difficult. Therefore, prior to measuring the maintenance force of dentures in the oral cavity, basic data must be collected first by performing measurements using an experimental palatal base in dentulous individuals with stable conditions. In a previous study [16] using a model, we investigated the factors influencing the measurement of the retentive force of the palatal plate, assuming an edentulous oral cavity. The results showed that the direction of traction and pressure load of the palatal plate influenced the measurement of retentive force. This study aimed to compare and investigate the retentive force of a palatal plate in the oral cavity brought by the use of denture adhesives for dry mouth, other conventional denture adhesives, and oral moisturizers, using two newly invented devices to define pressure load and traction direction.

## Methods

### Test samples (Table 1)

**Table 1:** Principal components of the test sample

Test samples	Abbreviation	Components	Purpose of use
Pitatto Kaiteki Gel®	PK	Sodium polyacrylate	Thickening agent
		Sodium hyaluronate	Water retention
		Sodium alginate	Thickening agent
		Glycerol esters of fatty acids	Emulsifier
		Propyl paraoxybenzoate	Antiseptic, Bacteriostatic action
		Propylene glycol	Moisturizer, Solvent
		Macrogol 400	Solvent
		Purified water	
New Poligrip®	NP	Na/Ca methoxyethylene maleic anhydride polymer	Adhesion
		White vaseline	Moisturizer
		Carboxymethyl cellulose	Thickening agent, Emulsifier
		Soft liquid paraffin	Emulsifier
		Propyl paraoxybenzoate	Antiseptic, Bacteriostatic action
Biotene Oral balance Jell®	BT	Glycerin	Sweetening materials, Moisturizer, Thickening agent
		Sorbitol	Alternative sweetener, Moisturizer
		Xylitol	Alternative sweetener
		Carbomer	Viscosity adjustment
		Hydroxyethylcellulose	Moisturizer, Thickening agent
Denture Wet®	DW	Squalane	Oil-based component
		Dextrin palmitate	Thickening agent
		Diisostearyl malate	Viscosity adjustment
		Olive fruit oil	Moisturizer
		Stearyl glycyrrhetinate	Solvent
		Menthol	

This study was a pilot controlled clinical trial of a novel denture adhesive for dry mouth (PK) with a water-soluble main ingredient. In this trial, PK was compared to other three active experimental conditions, including a traditional denture adhesive and two types of oral moisturizers. A negative control condition (fifth condition) was also added to the study. In this study, the following test samples were prepared: denture adhesive for dry mouth (PK; Pitatto Kaiteki Gel®: NISIKA, Yamaguchi, Japan), cream-type denture adhesive (NP; New Poligrip® Sa: Glaxo Smith Kline, Tokyo, Japan), gel-type oral moisturizer (BT; Biotene Oral balance Jell®: T&K, Tokyo, Japan), denture moisturizer (DW; Denture Wet®: DENTCARE, Osaka, Japan). The amount of each coating was 1.5 g for PK, 0.6 g for NP, 1.8 g for BT, and 1.2 g for DW.

### Participants

The study participants included 10 healthy adult dentulous individuals (5 males and 5 females, mean age 27.2 ± 1.6 years) who fully understood the purpose of the experiment and provided written consent. The exclusion criteria of this study were as follows: those with intraoral findings that would interfere with measurements (e.g., torus palatinus or oral mucositis), those with a vomiting reflex, and those undergoing medication therapy. However, none of the 10 participants met the above exclusion criteria; thus, all were included in the final study cohort. First, impressions of the maxillae of the 10 participants were taken. A palatal plate was fabricated on a plaster cast made from an impression, and the retentive force was measured under five conditions, including the use of four test substances and a control without any application. In addition, a subjective evaluation of the usability of the four test substances was conducted using a questionnaire. Each of the five conditions was evaluated on a different day.

### Fabrication of the palatal plates

**Fig. 1:**
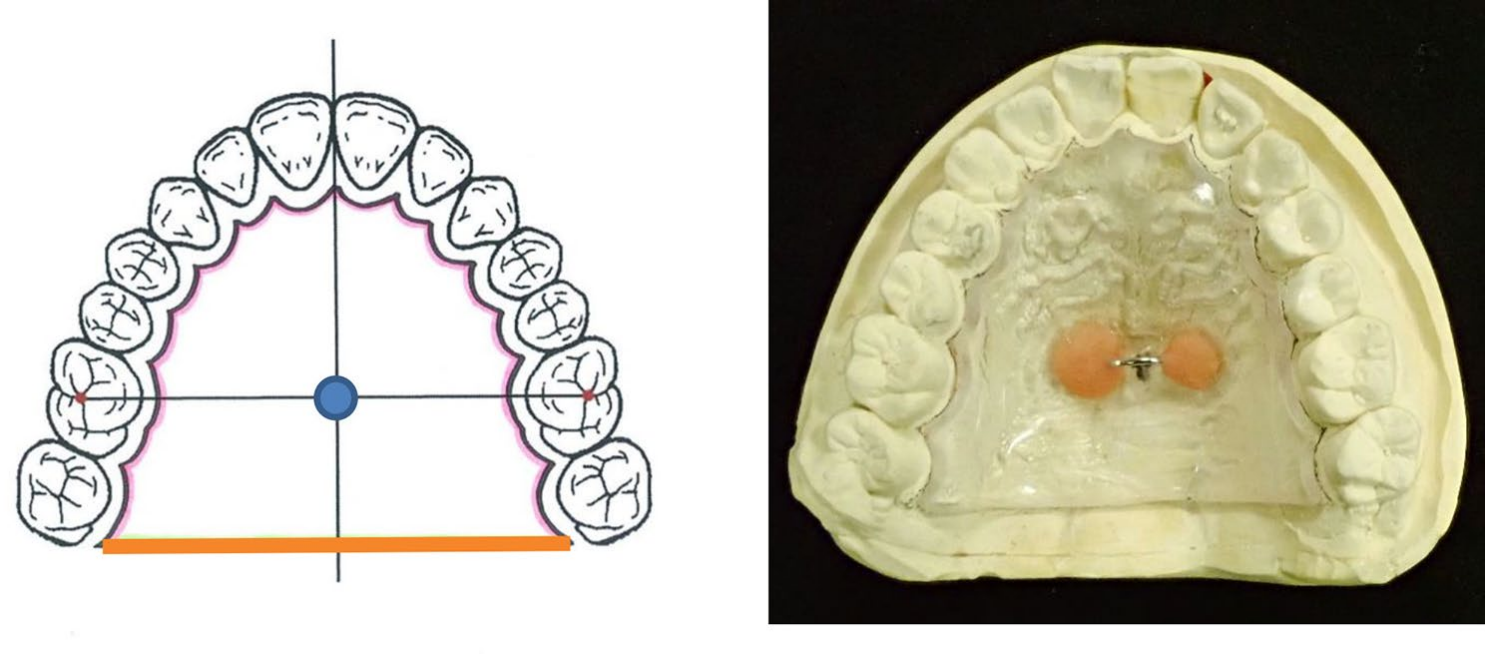
Working cast and palatal plate The palatal plate was fabricated using a 3.0-mm thermoplastic resin sheet. A traction ring was added to the centre of the plate.

Impressions of the maxillary dentition of the 10 study participants were taken using alginate impression material (AROMA FINE PLUS®; GC, Tokyo, Japan). Models for fabrication of the palatal plates were made from improved dental stone (NEW FUJIROCK®; GC, Tokyo, Japan). The design of the palatal plates was similar to the model used in a previous study [16]. A 3.0-mm thick thermoplastic sheet was formed on a plaster cast using a heat moulding machine (Ercopress®; Ercodent Erich Kopp Gmbh, Germany) to fabricate a palatal plate. The palatal plate was set 1 mm medial to the palatal cervix of the maxillary dentition, and the posterior margin of the plate was set on a straight line connecting the distal surfaces of the bilateral second molars. Traction rings were made using 0.9-mm diameter Co-Cr alloy wire (Sun-Cobalt Clasp- Wire®; Dentsply Sirona, Tokyo, Japan) and placed at the centre of each palatal plate at the intersection of the midline and the straight line connecting the central fossa of the bilateral first molars (Fig. 1). The traction ring was fabricated as a semicircle with a radius of 4.0 mm to fit the tip of the retentive force measurement device.

### Retentive force measuring device

**Fig. 2:**
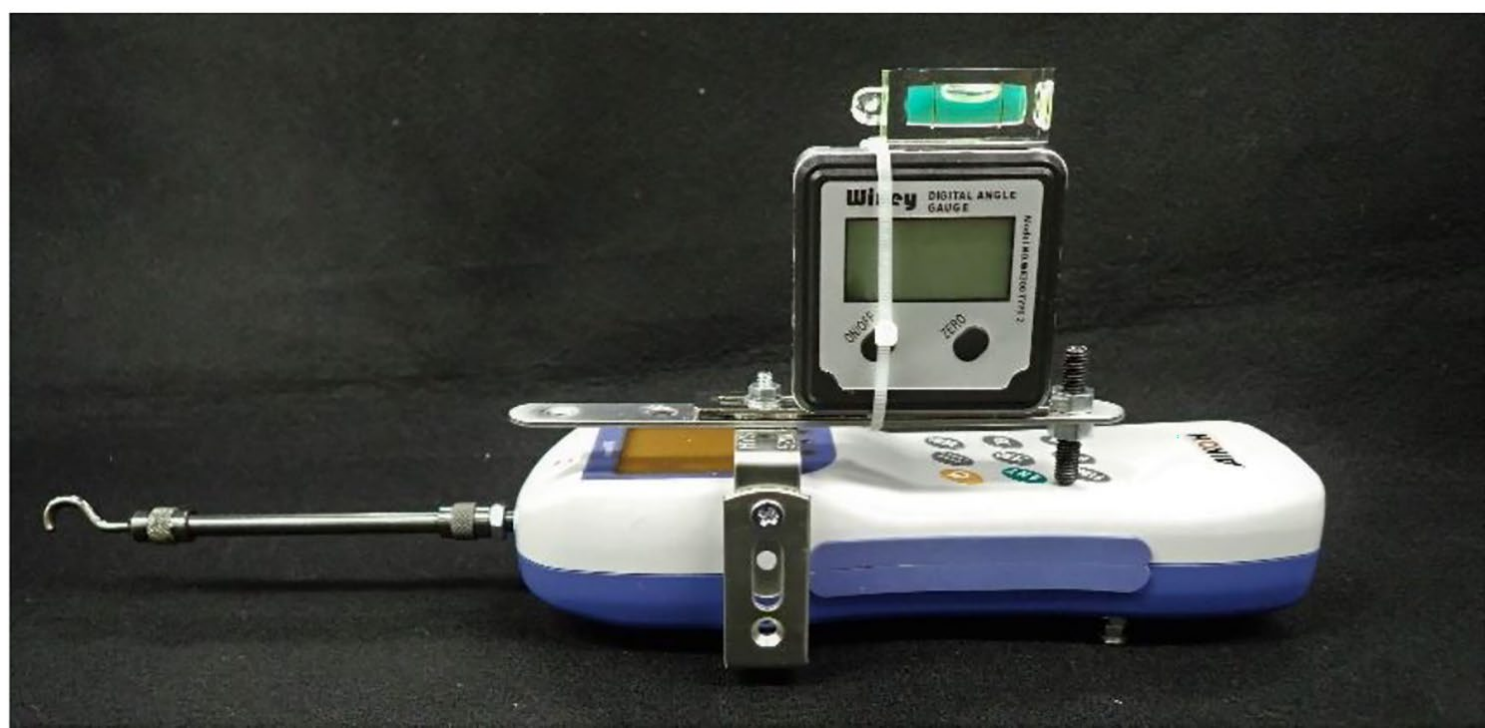
The measuring device with angle meter attached Measuring the retention of the palatal plate by hooking the tip of the device (Digital force gauge RZ-5®; AIKOHENGINEERING, Tokyo, Japan) to a ring and applying traction. The angle meter (Digital angle gage WR300 Type 2®, Wixey, US) displays the direction of traction relative to the Camper’s plane.

With reference to previous studies [11, 15, 16], a digital force gauge (RZ-5®, AIKOHENGINEERING, Tokyo, Japan) was used to measure the retentive force of the palatal plate. A retrofittable measuring hook (measuring attachment number, 011 B; AIKOHENGINEERING, Tokyo, Japan) was attached to the tip of the digital force gauge after morphological modification to allow traction on the palatal plate. The body of the digital force gauge was fitted with an angle gauge (Digital angle gage WR300 Type 2®, Wixey, US) that displayed the inclination of the device to define the traction direction of the palatal plate (Fig. 2). The virtual line connecting the superior border of the tragus and the inferior border of the ala of the nose (Camper’s plane) was used as a reference during the measurements. Traction was applied at an angle of 60° from the reference plane.

### Fabrication of the devices for pressure welding

**Fig. 3:**
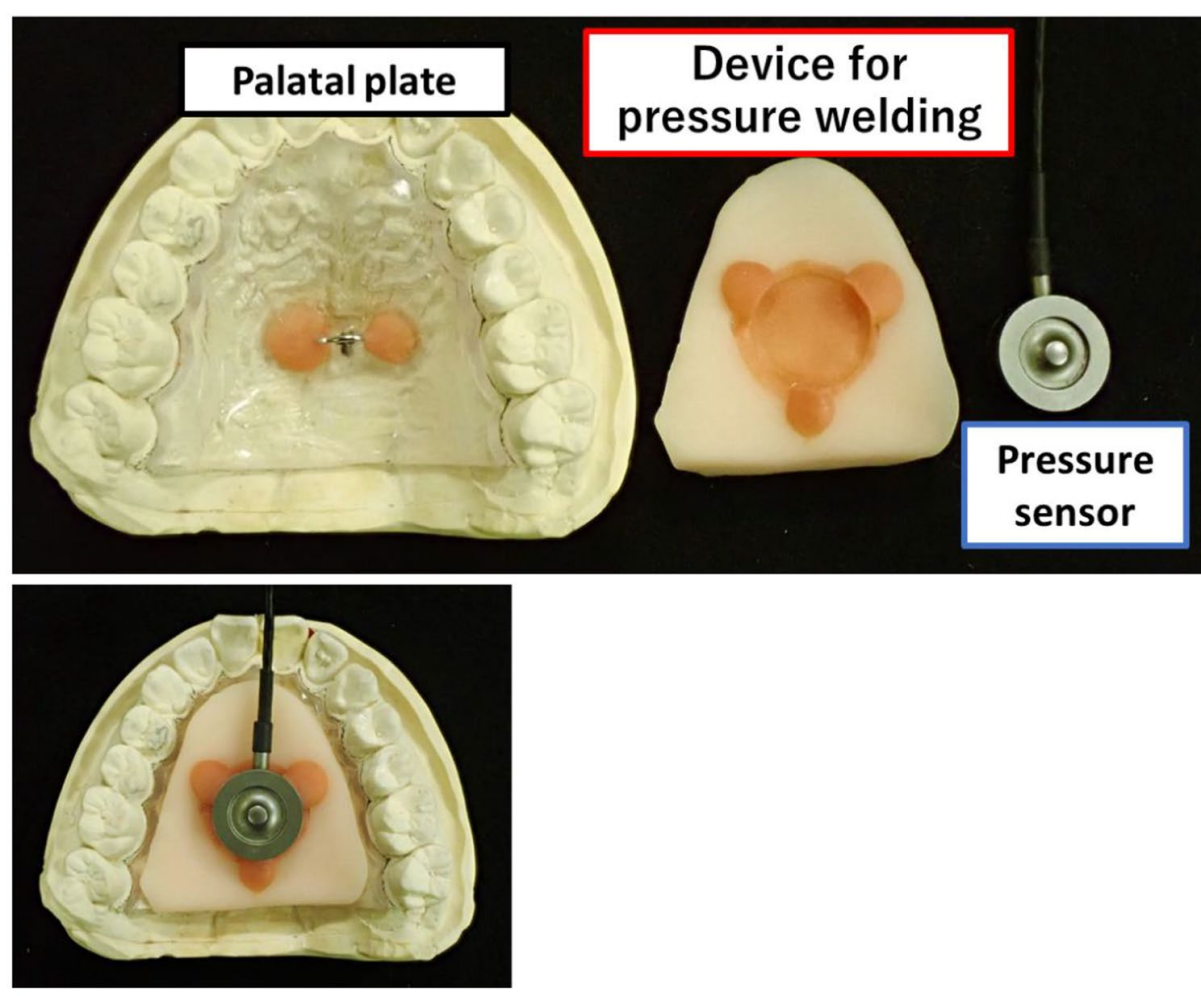
The device for pressure welding was fabricated to fit the palatal plate of each of the 10 study participants. The device was also moulded such that a pressure sensor could be integrated into it.

Previous studies have shown that the load on the palatal plate during pressure contact influences the retentive force. For this reason, a device was needed to define the loads. We fabricated palatal plates that were seated under standardized pressures using custom-made devices for pressure welding. In turn, the retentive force was measured using a digital force gauge at specific periods. The devices were made to maintain a constant pressure load with the placement of the palatal plate in the mouth and application of pressure. These devices for pressure welding were fabricated for each of the 10 palatal plate surfaces by moulding room temperature-cured resin (TrayResin®, Shofu, Kyoto, Japan). The pressure sensor (compact pressure contact load cell LMA-A®, Kyowa, Tokyo, Japan) was incorporated into the pressure welding device (Fig. 3).

### Measuring conditions

The five measurement conditions were: (1) control, storage in water only and no test sample applied; (2) NP coating; (3) PK coating; (4) BT coating; and (5) DW coating. The order by which the five conditions were measured was determined using the Latin square design. The study participants were instructed to rinse before the start of the measurements and sit during the measurements. The measurements started at 16:00. In all cases, oral moistness was measured with an oral moisture meter (Mucus®; LIFE, Saitama, Japan) prior to the measurements to ensure that there was no dry mouth.

### Measurement of retentive force

**Fig. 4:**
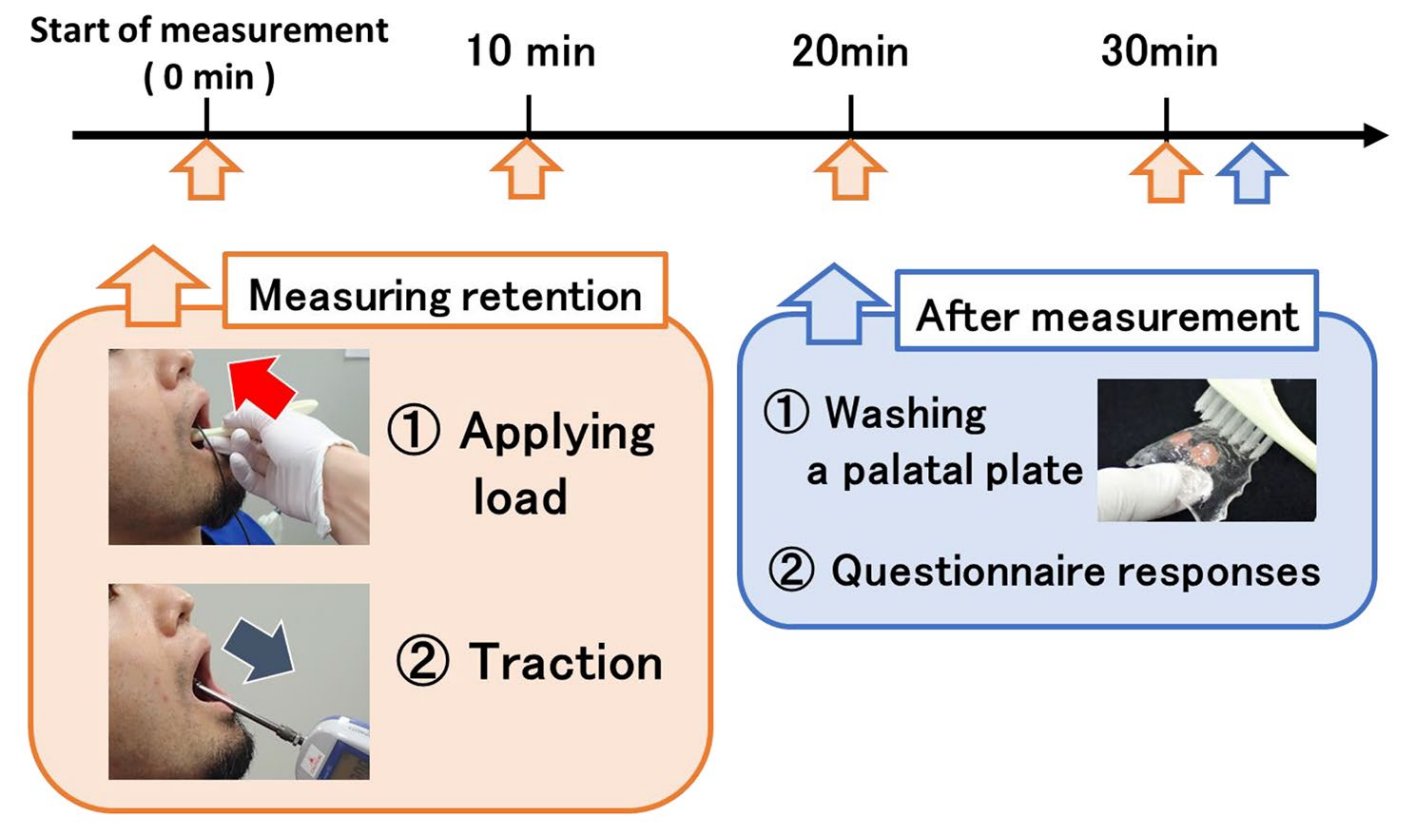
The sample was applied to the inner surface of the floor of the palatal plate. Afterwards, the palatal plate was placed in the mouth for 10 s at 25-N pressure. The pressure sensor was then retracted, and the retention force was measured. After measuring the holding force, the plate was again pressed into the mouth. Measurements were taken every 10 min for 30 min, starting when the floor of the palate was placed. Measurements were repeated four times at each time point. After the measurements were completed, the study participants were asked to wash the palatal plate with water and complete a questionnaire on their evaluation of the samples used.

The palatal plate was placed in the mouth after applying the substance to its inner surface. Then, a constant load of 25 N was applied for 10 s in the direction of the occlusal plane using the device for pressure welding. The palatal plate was then towed at an angle of 60° to Camper’s plane at a rate of 1 N/s to measure the retentive force. The maximum retentive force until the palatal plate detached from the palatal mucosa was measured. After measurement, the palatal plate was again fitted, and 25 N pressure was applied using the device for pressure welding. The measurements started when the palatal plate was fitted and were taken every 10 min for 30 min. They were repeated four times at each 10-min time point, with the first time being excluded and the average of the second to fourth times taken as the measured retentive force at that time point. In addition, averages were calculated for each time point for the 10 study participants (Fig. 4).

### Subjective evaluation of the usability of the test sample

After the measurement of retentive force, the study participants were asked to rinse the palatal plate with water and evaluate five parameters—taste, unstickiness, stability, wettability, and washability—on a 100-mm visual analogue scale after each test sample was used (Fig. 4). For the three criteria of ‘taste’, ‘stability’, and ‘wettability’, 0 mm was considered ‘very bad’, while 100 mm was considered ‘very good’. For the item ‘unstickiness’, 0 mm was considered ‘very strong stickiness’, while 100 mm was considered ‘very weak stickiness’. For ‘washability’, 0 mm was considered as ‘very difficult to wash’, while 100 mm was considered as ‘very easy to wash’.

### Statistical analysis

Statistical analysis was performed using IBM SPSS Statistics for Windows, version 27 (IBM Corp., Armonk, N.Y., USA). A one-way analysis of variance was performed for each test substance at each time and for each substance at each time point. Tukey’s method was then used to analyse whether each test substance changed over time and whether there was a difference in retentive force between substances at each time point. A one-way analysis of variance was performed on the results of the subjective evaluation of usability. Tukey’s method was used to analyse whether there was a difference in usability among the substances for each question item. All significance levels were set at 5%. The null hypotheses were that the retentive force does not change with time, that the retentive force changes with the type of substance, and that the usability does not change with the type of substance.

## Results

### Retentive force

#### Comparison of the retentive force for test samples at each time point (Figs. 5 and 6) (Table 2)

PK, NP, and BT showed significantly higher retentive force than the control immediately after application (p < 0.05). No significant difference was observed between DW and the control (p < 0.05). After 10 min of application, PK showed higher retentive force than the control (p < 0.05). NP showed the highest retentive force among the control and test substances (p < 0.05). BT and DW were comparable to the control (p < 0.05). After 20 min of application, PK showed higher retention than the control (p < 0.05). Similar with the 10-min duration, NP showed the highest retentive force among the control and test substances (p < 0.05). BT and DW were also comparable to the control (p < 0.05). After 30 min of application, only NP showed a higher retentive force than the control, PK, and BT. DW showed the highest retentive force among the test substances (p < 0.05). BT and DW were comparable to the control (p < 0.05).

**Table 2:** Measuring retentive force (N)

	Control	PK	NP	BT	DW
Start of measurement	0.84 ± 0.45	2.97 ± 0.98	2.34 ± 1.06	2.26 ± 0.51	1.60 ± 0.68
10 min	0.89 ± 0.37	2.57 ± 0.57	5.00 ± 0.80	1.58 ± 0.57	1.64 ± 0.76
20 min	0.90 ± 0.39	2.23 ± 0.58	5.99 ± 1.38	1.31 ± 0.55	1.51 ± 0.55
30 min	0.89 ± 0.41	1.98 ± 0.64	7.32 ± 2.85	1.19 ± 0.40	1.57 ± 0.75

#### Changes in retentive force in each test substance over time (Figs. 5 and 7) (Table 2)

There was no significant difference in the retentive force of the control and DW immediately after application and after 10, 20, and 30 min (p < 0.05). PK showed significantly lower retentive force after 30 min than immediately after application (p < 0.05). Moreover, no significant differences were found among the retentive forces immediately after application and at any time point, except at 30 min (p < 0.05). NP has significantly lower retentive force immediately after application than after 10, 20, and 30 min; however, there were no significant differences among the other time points (p < 0.05). BT has significantly higher retentive force immediately after application than after 10, 20, and 30 min; however, there were no significant differences among the other time points (p < 0.05).

### Subjective evaluation of the usability of the test substance (Table 3; Fig. 8)

In the ‘taste’ parameter, BT showed significantly higher values than the other three test samples, and there were no significant differences among the other test samples (p < 0.05). NP was lower than PK, BT, and DW in the ‘unstickiness’ parameter (p < 0.05). Meanwhile, DW showed lower values than BT (p < 0.05). PK was higher than DW in the ‘stability’ parameter (p < 0.05). Meanwhile, NP was higher than BT and DW (p < 0.05). No significant difference was observed between DW and BT (p < 0.05). In the ‘wettability’ parameter, PK was higher than NP but lower than BT (p < 0.05). BT was the highest among the test samples (p < 0.05). Finally, PK was higher than NP and DW in the ‘washability’ parameter (p < 0.05). Meanwhile, no significant difference was observed between PK and BT (p < 0.05). NP showed lower values than PK and BT (p < 0.05). DW was the lowest among the test samples (p < 0.05).

**Table 3:** Subjective evaluation of the usability of the test samples (mm)

	Taste	Unstickiness	Stability	Wettability	Washability
PK	32.4 ± 18.4	71.7 ± 24.5	72.3 ± 15.5	66.6 ± 17.2	96.0 ± 3.57
NP	31.7 ± 16.2	5.85 ± 4.52	78.8 ± 21.2	40.6 ± 12.0	53.1 ± 29.9
BT	75.6 ± 11.46	88.6 ± 15.1	49.6 ± 16.7	86.2 ± 9.52	95.4 ± 2.95
DW	43.4 ± 28.6	53.5 ± 27.8	33.6 ± 28.9	53.2 ± 14.1	28.7 ± 23.2

## Discussion

In this pilot trial, we compared a novel denture adhesive for dry mouth (PK) to an array of other conditions. The other four tested conditions were conventional cream-type denture adhesive, oral moisturizer, denture moisturizer with an oil-based ingredient, and a negative control. In general, PK showed promising results in terms of achieved retentive strength over time and subjective outcomes.

In this study, BT, which has a high viscosity, was selected among the oral moisturizers. Yamagaki et al. [7] reported that oral moisturizers with high viscosity showed the same retentive force as denture adhesives. Meanwhile, DW was selected as the representative of oil-based moisturizers. As preliminary experiments showed that different amounts of coating had an influence on the retentive force, a defined amount of test substance was used. NP was weighed according to the instructions on the package insert regarding the recommended dosage. Eventually, 0.6 g of the substance was applied. For PK, 1.5 g was applied according to the instructions on the package insert. As the instruction manuals for BT and DW did not indicate the recommended amount for dentures, we weighed them according to the same volume as PK (1.5 g), since they are the same gel-like material and have similar physical properties. Finally, 1.8 and 1.2 g of BT and DW, respectively, were applied.

Regarding the testing procedures, used devices followed standards set by a previous study, starting by the retentive force measuring device. To define the direction of traction, the angle gauge used in this study (Digital angle gage WR300 Type 2®, Wixey, US) can be set with reference to any plane. First, the arm at the end of the maintenance force measuring device was used to align the reference to the Frankfort plane of the participant, from which the device was tilted to 60° for traction. The use of the Camper’s plane as the reference plane in setting the virtual occlusal plane seems reasonable since it is used in daily clinical practice. In a study by Bandai et al. [17], wherein the retentive force of the palatal plate was measured, the traction direction was also defined with reference to the Camper’s plane.

This study also standardized the time for experimental procedures. Considering the diurnal variation of salivary secretion and body temperature [18, 19], the starting time of the measurements was set at 16:00. In the model experiment of a previous study, the first measurement after the pressure contact showed the largest variation in values when measuring the retentive force. In the present study, the measurements after the second one, when stable values can be obtained, were used.

The two null hypotheses, that retentive force does not change with time and that the retentive force does not change with the type of substance, were rejected by one-way ANOVA. PK, NP, and BT showed higher retentive force than the control immediately after fitting. On the other hand, no significant difference was observed between DW and controls. Therefore, DW may not be able to increase retentive force. After 10 min, the retentive force of BT decreased to the same level as that of the control, suggesting that the duration of the effect of BT on the palatal plate was approximately 10 min only. BT contains more water-soluble components such as glycerol, which may have been washed out from the mucosal surface by saliva, resulting in a shorter duration than that of NP. After 20 min of application, NP showed the highest retentive force. PK showed higher retentive force than the control, but lower than NP. Since NP binds to water and exhibits adhesive properties, it became compatible with the water in the oral cavity in approximately 20 min, which generated a strong retentive force. A significant difference was observed after 30 min, suggesting that its effect lasts from 20 to 30 min, compared with the control. However, the retentive force decreased over time, so it could be necessary to apply again after 30 min of use. NP remained to have high retentive force, suggesting that it is effective in increasing the retentive force for more than 30 min. PK showed a gradual decrease in retentive force over time. Meanwhile, NP showed a rapid increase in retentive force from immediately after the application to 10 min afterwards. Compared with the other test substances, 10 min is necessary for it to take effect and for the retentive force to stabilise. When using BT, the retention force was stronger immediately after application than at other time points, and decreased after 10 min. Therefore, its duration of effect was within 10 min, and its effectiveness as a substitute for denture adhesives was limited. Finally, DW did not show significant differences over time, suggesting that it is unlikely to be effective in increasing retentive force.

In terms of subjective properties, the null hypothesis that the usability does not change when the sample is changed was rejected by one-way ANOVA. PK performed relatively well for wettability and washability, which can be explained by its moisturizing ingredients (e.g., sodium polyacrylate and sodium hyaluronate). Due to its higher moisture retention, its ‘wettability’ is higher in the subjective evaluation. Its ‘washability’ is also higher due to its high content of water-soluble components. A report by Ohno et al. [14], wherein the time taken for a substance to run off under running water was measured, showed that PK could be flushed in a shorter time compared to NP. NP is considered to have low ‘unstickiness’ and ‘washability’ because Na/Ca methoxyethylene maleic anhydride polymer produces strong adhesion. However, its ‘stability’ was high. NP also absorbs water and become sticky, hence it reduced ‘wettability’. BT contains substitute sweeteners such as xylitol, which may have led to its higher evaluation in ‘taste’. It also contains water-soluble moisturizing components such as glycerin as its main ingredient, which may have led to its higher ratings in ‘wettability’ and ‘washability’. DW had the lowest ability to increase retentive force, which also resulted in the lowest subjective evaluation of ‘stability’. Furthermore, it had the lowest ‘washability’ rating, possibly because its oily component, squalane oil, made removal by rinsing difficult.

The limitations of the present study include taking measurements from healthy study participants without dry mouth and subjects having stable oral environmental conditions. Furthermore, this measurement time was 30 min, which was assumed to be the duration of one meal, but the effect of a longer using was not clear. A study by Ohno et al. [10] showed that in a model of severe dry mouth, the denture adhesive for dry mouth produced greater retentive forces than the conventional cream-type denture adhesive. However, saliva possibly had less effect on the substance in a dry oral cavity. Based on the retentive force measurement on the palatal plate and evaluation of the usability in this study, PK may be effective for daily use because it is easy to wash with water, while NP may be used when a long-lasting effect is required, such as when going out. However, it is still unclear if this is strictly true for actual denture use. Furthermore, the effect of occlusal pressure on the denture during eating may also influence retentive force. Thus, future studies must consider the differences in conditions such as jaw crest morphology and dry mouth and make measurements in actual denture wearers, especially those with dry mouth.

## Conclusions

The onset of effect of the denture adhesive for oral dryness was rapid, suggesting that it was effective for about 20 min, even in oral cavities without dryness. Its stabilising effect was comparable to that of the conventional cream-type denture adhesive. Finally, it had good cleaning and other usability characteristics.

**Fig. 5:**
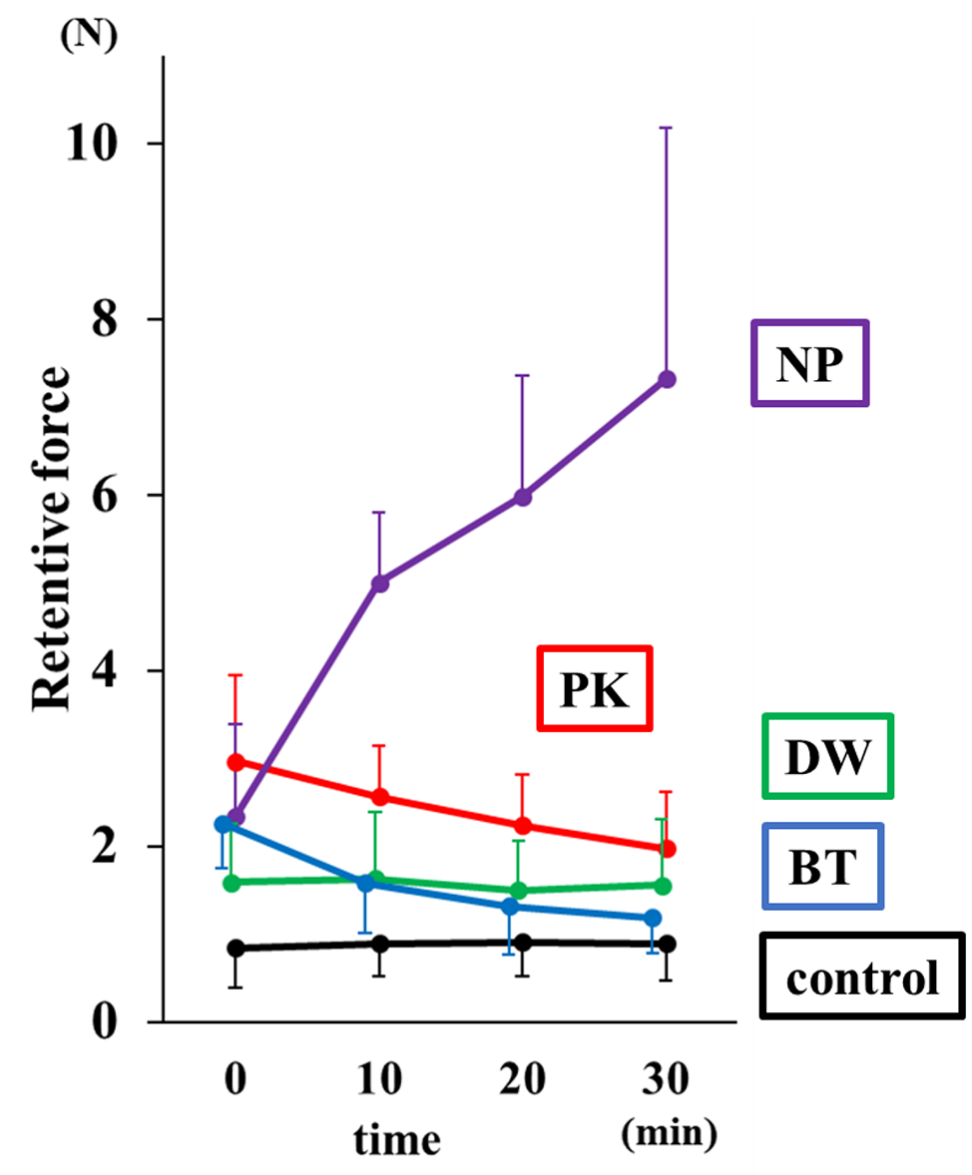
Measuring the retentive force

**Fig. 6:**
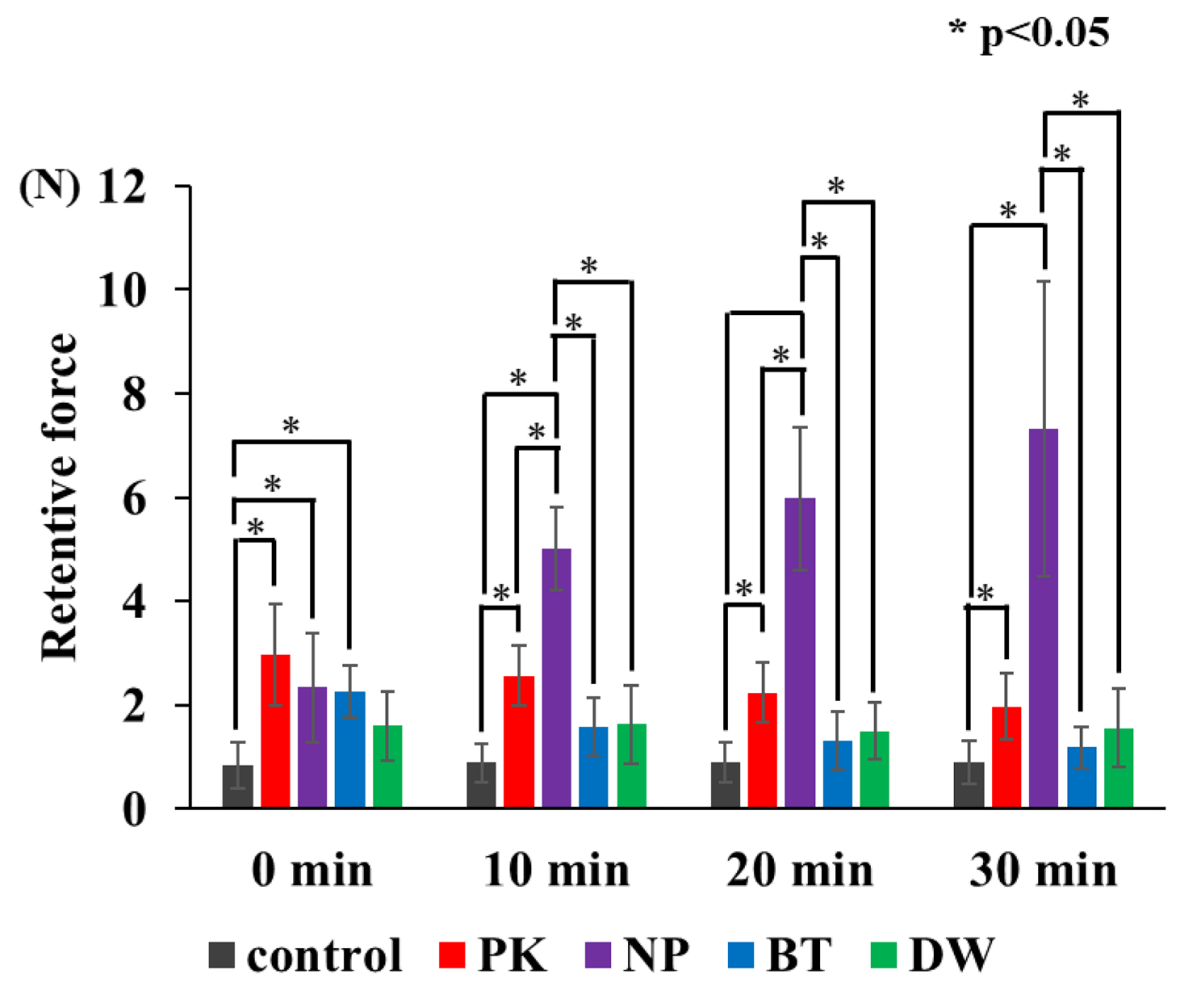
Comparison among the test samples at each time

**Fig. 7:**
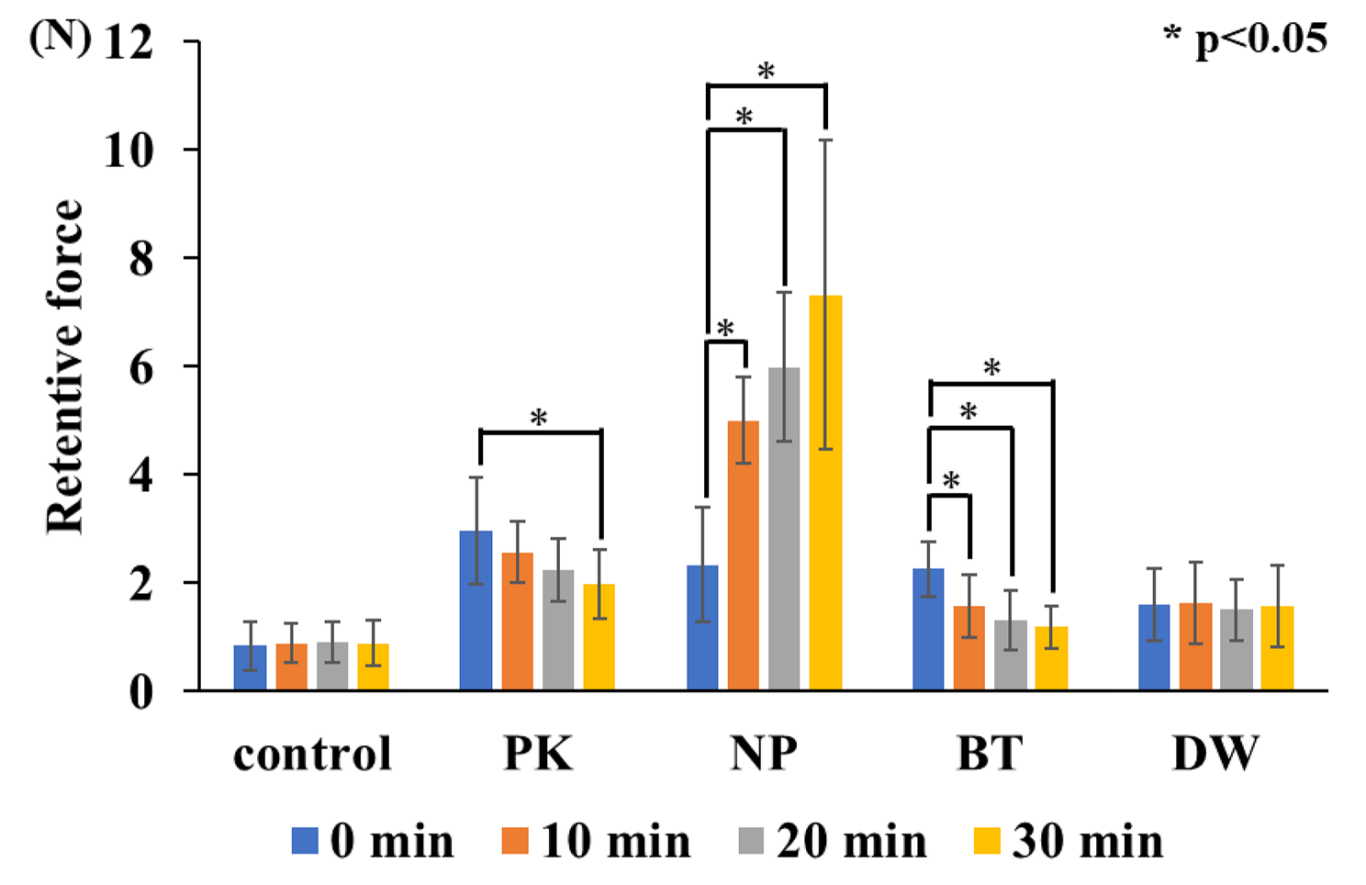
Changes over time for each test samples

**Fig. 8:**
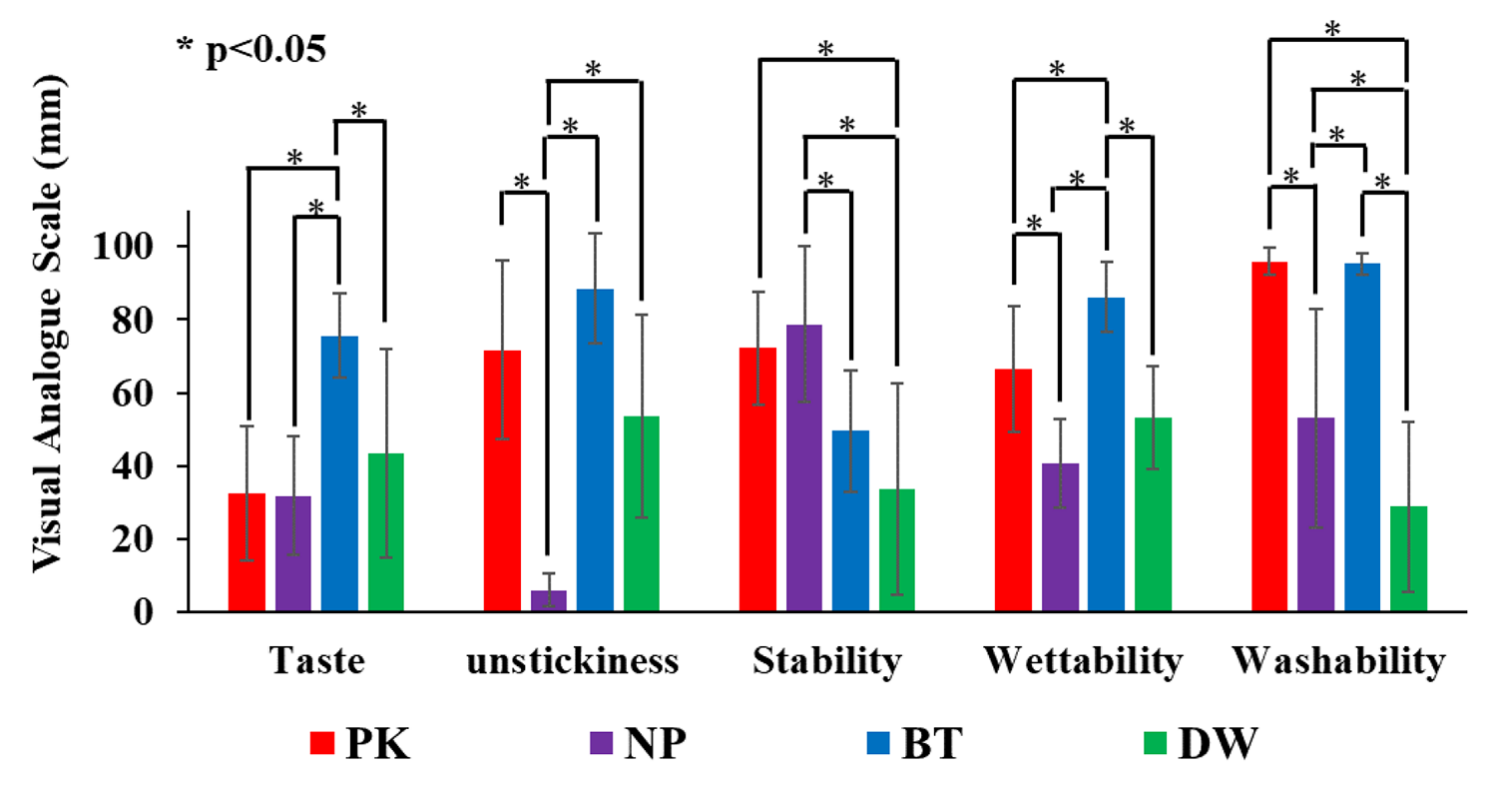
Subjective evaluation of the usability of the test samples

## Data Availability

The datasets generated and/or analysed during the current study are not publicly available due to protect the privacy of study participants but are available from the corresponding author on reasonable request.

## Abbreviations

NP: cream-type denture adhesive
PK: gel-type denture adhesive for dry mouth
BT: gel-type oral moisturizer
DW: denture moisturizer
